# Relationship between Anti-Spike Antibodies and Risk of SARS-CoV-2 Infection in Infants Born to COVID-19 Vaccinated Mothers

**DOI:** 10.3390/vaccines10101696

**Published:** 2022-10-11

**Authors:** Madeleine D. Burns, Cordelia Muir, Caroline Atyeo, Jameson P. Davis, Stepan Demidkin, Babatunde Akinwunmi, Alessio Fasano, Kathryn J. Gray, Galit Alter, Lydia L. Shook, Andrea G. Edlow, Lael M. Yonker

**Affiliations:** 1 Mucosal Immunology and Biology Research Center, Boston, MA 02114, USA; 2 Department of Pediatrics, Massachusetts General Hospital for Children, Boston, MA 02114, USA; 3 Vincent Center for Reproductive Biology, Massachusetts General Hospital, Boston, MA 02114, USA; 4 Ragon Institute of Massachusetts General Hospital, Massachusetts Institute of Technology and Harvard, Cambridge, MA 02139, USA; 5 Harvard Medical School, Boston, MA 02115, USA; 6 Division of Maternal-Fetal Medicine, Department of Obstetrics and Gynecology, Brigham and Women’s Hospital, Boston, MA 02115, USA; 7 Division of Maternal-Fetal Medicine, Department of Obstetrics and Gynecology, Massachusetts General Hospital, Boston, MA 02114, USA

**Keywords:** COVID-19, humoral immune response, vaccines, pediatrics, pregnancy, transplacental antibody transfer

## Abstract

The goal of this study was to investigate the relationship between anti-SARS-CoV-2-Spike IgG titers passively transferred to the fetus from maternal vaccination during pregnancy and timing of infant SARS-CoV-2 infection. Pregnant, vaccinated individuals (n = 105) and their infants (n = 107) were enrolled in a prospective cohort study from July 2021 to June 2022, linking infant anti-Spike IgG titer at birth to risk of SARS-CoV-2 infection in the first fifteen months of life. Cord blood sera were collected at delivery and infant sera were collected at two and six months of age. Anti-SARS-CoV-2-Spike IgG levels were quantified in cord and infant sera using an enzyme-linked immunosorbent assay. Infants were followed for SARS-CoV-2 infection through fifteen months of age. Anti-SARS-CoV-2-Spike IgG titers in infants declined significantly with increased age (*p* < 0.001). Infants with higher anti-Spike cord blood levels had significantly longer disease-free intervals prior to infection with SARS-CoV-2 (*p* = 0.027). While higher anti-Spike IgG titer at two months of age was associated with a longer interval to infection through nine months of age (*p* = 0.073), infant anti-Spike IgG titers by six months of age had no impact on disease-free interval. This cohort study suggests that passively transferred maternal IgG is protective against infant SARS-CoV-2 infection, with higher antibody levels at birth significantly associated with longer disease-free intervals. Infant antibodies and protection from SARS-CoV-2 infection wane significantly after six months, suggesting that vaccination is needed at this stage to optimize protection against COVID-19.

## 1. Introduction

The Coronavirus Disease 2019 (COVID-19) pandemic poses a significant risk to infants [1,2] who rely on acquired maternal immunity during the first months of life for protection from disease [3]. Maternal Severe Acute Respiratory Syndrome Coronavirus 2 (SARS-CoV-2) vaccination in all trimesters of pregnancy has been shown to protect the maternal-fetal dyad via the generation and passive transplacental transfer of anti-Spike IgG antibodies, which can be detected in the umbilical cord at birth [4,5]. Although maternal COVID-19 vaccination in pregnancy reduces the risk of hospitalization in SARS-CoV-2-infected infants [6,7] and passively transferred vaccine-induced immunoglobulins against SARS-CoV-2 persist in neonatal blood for up to six months of age [8], the relationship between anti-Spike IgG titers in infants after maternal vaccination and their role in protection against neonatal SARS-CoV-2 infection has not been defined. Anti-SARS-CoV-2 antibodies have been demonstrated to be correlates of protection against severe COVID-19 in adults [9,10,11] and children [12,13,14], reducing the severity of disease and limiting complications such as Multisystem Inflammatory Syndrome in Children [15]. Given that infants under six months of age are not yet eligible for COVID-19 vaccination, it is critical to understand the degree of protection provided to infants following birth by maternal immunization during pregnancy.

This prospective cohort study characterizes the serologic and clinical trajectories of 107 infants born to COVID-19 vaccinated pregnant individuals, filling a missing link between the detection of anti-SARS-CoV-2 titers in neonates and infants after maternal vaccination and the protection these antibodies might provide infants against SARS-CoV-2 infection and severe disease.

## 2. Materials and Methods

### 2.1. Human Subjects

Pregnant individuals receiving care at Massachusetts General Hospital or Brigham and Women’s Hospital 18 years or older who completed their full-dose primary vaccine series (two doses of either Pfizer (BNT162b2) or Moderna (mRNA-1273), or one dose of the Johnson & Johnson vaccine (Ad26.COV2.S)) during pregnancy were identified and approached for informed consent to participate in a prospective biorepository study (MGB IRB #2020P003538). Infants were enrolled with parental consent while in utero (at time of pregnant individual consent) or after delivery to participate in a follow-up study associated with the MGH Pediatric COVID-19 Biorepository (MGB IRB #2020P000955) [16] from July 2021 to June 2022. Cord sera was collected at time of maternal delivery and capillary sera was collected from infants via microneedle device (YourBio Health, Medford, MA, USA) at two or six months of age. Families had the opportunity to decline sample collection at any point. Clinical metadata were extracted from the electronic health record.

### 2.2. ELISA

The anti-Spike IgG levels described in these analyses have been included in a prior manuscript [8], but the titers have not previously been correlated with SARS-CoV-2 infectious outcomes in infants through 15 months of age. Anti-SARS-CoV-2-Spike IgG titers were quantified using an enzyme-linked immunosorbent assay (ELISA), as previously described [8]. In brief, ELISA plates were coated with 500 ng/mL of D614G-Spike and incubated at room temperature for 30 min. Plates were washed then blocked with a 0.1% BSA solution. Sample was added (1:100 dilution) and plates were incubated at 37 °C for 30 min prior to washing. A horseradish peroxidase (HRP)-conjugated goat anti-human, Spike-specific IgG antibody (Bethyl Laboratories, Montgomery, TX, USA) was added, incubated for 30 min, washed, then the reaction was stopped. Signal was read at 450 nm and PBS-background corrected from a reference wavelength of 570 nm. Detectable Anti-Spike IgG was defined as any value greater than the sum of the mean value of assay negative controls (known SARS-CoV-2 negative, unvaccinated individuals) and 3 × the standard deviation of those samples.

### 2.3. Clinical Outcomes

All parents completed a survey regarding positive or negative infant histories of SARS-CoV-2 infection and any associated symptoms and treatments upon completion of the study. If positive, parents were asked to report method of diagnosis (at-home rapid antigen test, clinically obtained PCR, or presumed diagnosis based on exposure and symptoms). SARS-CoV-2 testing was performed at the discretion of the parents. COVID-19 severity was determined based on NIH criteria [17].

### 2.4. Analysis

Simple linear regression was used to demonstrate the inverse relationship between anti-Spike IgG levels and infant age. Kaplan–Meier survival curves were used to show infection-free time intervals based on upper and lower quartiles of anti-SARS-CoV-2-Spike IgG levels in cord sera, infant capillary sera at two months of age, and infant capillary sera at six months of age. Significant differences between infection-free intervals in infants with the top and bottom quartiles of anti-SARS-CoV-2-Spike IgG were assessed by a Mantel-Cox Test. Fisher’s Exact test and Mann–Whitney U test were used to determine significant differences between groups (SARS-CoV-2-infected vs. uninfected infants) with respect to neonatal sex, gestational age at delivery, birthweight and breastfeeding status at time of infection or study completion. All analyses were conducted using Graphpad Prism version 9.0.

## 3. Results

While vaccine-generated antibodies against SARS-CoV-2 have been shown to transfer across the placenta from mother to fetus [8,18,19], the clinical impact of this passive transfer has not been directly evaluated. Descriptive data for the 105 pregnant individuals enrolled are provided in Table 1. Fifty-nine percent (n = 62) of these individuals received the Pfizer (BNT162b2) vaccine, whereas 32.4% (n = 34) received the Moderna (mRNA-1273) vaccine and 8.6% (n = 9) the Johnson & Johnson (Ad26.COV2.S) vaccine. Six (5.7%) of the vaccinated pregnant individuals had a history of SARS-CoV-2 infection during pregnancy, 3 in first trimester, 1 in second trimester, and the remaining 2 in the third trimester. The majority of our cohort self-identified as White (n = 90, 85.7%), and 5.7% (n = 6) identified as Hispanic. From this maternal cohort, 107 infants, including two sets of twins, were enrolled in our neonatal study. Thirty-eight infants tested positive for SARS-CoV-2 throughout the 15-month follow-up period while 69 infants remained clinically uninfected at study completion (Table 1). There were no significant differences between infected and uninfected infants with respect to neonatal sex, gestational age at delivery, birthweight, or breastfeeding status at time of infection or study completion between the two groups (Table 1).

Infant symptoms of COVID-19 reported by parents were mild, the most common being congestion and rhinorrhea (86.8%), cough (65.8%) and fever (57.9%) (Table 2). Of the infants who developed COVID-19 in our study, none were hospitalized. Roughly half of infants did not require treatment (47.4%), while others managed symptoms at home using antipyretics (47.4%) and two required oral steroids for outpatient management of wheezing (5.3%). Over half (n = 21, 55.3%) of the infants had PCR testing to confirm infection, while 39.5% (n = 15) used only rapid antigen tests at home. Two infants were never tested but were symptomatic and presumed positive after exposure to a close household contact with confirmed, symptomatic COVID-19. The mean age of infants at time of infection was 8.5 months (SD 3.0 months) (Table 2).

**Figure 1:**
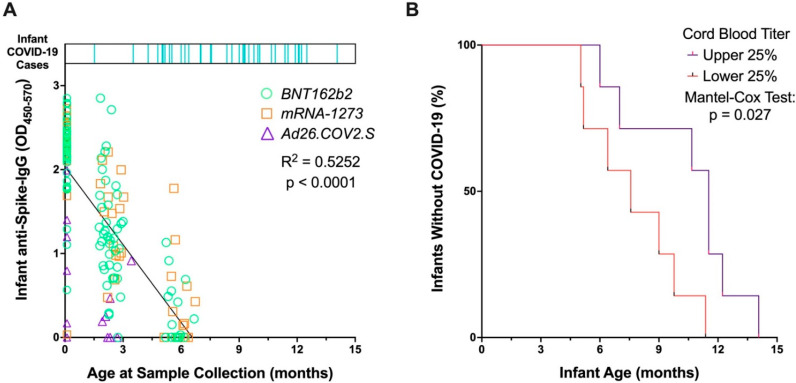
Relationship between infant anti-Spike titers from maternal vaccination and infant infection with SARS-CoV-2. (**A**) Timeline of COVID-19 onset in infants are shown in top bar, with each infant who developed COVID-19 depicted by a vertical blue line, with line position along the *x*-axis indicating age at SARS-CoV-2 infection. Bottom section displays infant anti-Spike IgG titers from maternal vaccination, measured in cord blood (age zero) and at two and six months of age. Units for infant anti-Spike IgG are OD_450–570_, which correspond to the optical density at 450 nm corrected from a reference wavelength of 570 nm. Symbols and colors are grouped by maternal vaccination platform: Green circles are for Pfizer (BNT162b2), orange squares for Moderna (mRNA-1273), purple triangles for Johnson & Johnson (Ad26.COV2.S). Analysis was performed by a simple linear regression. (**B**) Kaplan–Meier curve displays time to SARS-CoV-2 infection of infants who developed COVID-19, grouped by those who displayed high anti-Spike IgG titers in cord blood at birth (upper quartile) and those who displayed low anti-Spike IgG titers in cord blood (lowest quartile). Infants who developed COVID-19 with anti-Spike IgG titers from cord blood in the top quartile are more likely to experience a longer infection-free interval than infants with titers in the bottom quartile (*p* = 0.027). Analysis between groups was performed by a Mantel-Cox test.

Anti-SARS-CoV-2-Spike IgG titers after maternal vaccination in pregnancy were analyzed in 92 infants at birth in cord blood sera, capillary sera collected at two months, and/or capillary sera collected at six months of age. Anti-Spike IgG titers from passive maternal immunity waned significantly as infants aged (Figure 1A, simple linear regression *p* < 0.0001), especially after six months of age regardless of vaccine type. Frequency of infant COVID-19 cases increased with increasing age and declining titers (Figure 1A). One infant tested positive for SARS-CoV-2 at 1.5 months, otherwise no other infants developed SARS-CoV-2 prior to three months of age. Seventy-six percent of infected infants (29/38) tested positive after six months of age (Figure 1A).

The degree of passive transfer of maternal antibodies to the fetus can vary, depending on timing of maternal vaccination, maternal immune function, antibody glycosylation, and placental function and receptor expression, among other factors [5,19,20]. Therefore, we sought to determine if there were differences in clinical outcomes for infants depending on antibody titers at time of delivery. We analyzed duration of time (in months) until infants were infected with SARS-CoV-2, comparing infants who had the highest quartile of anti-Spike IgG in cord blood to those with anti-Spike IgG levels in the lowest quartile. Of those infants who ultimately tested positive for SARS-CoV-2, those with higher anti-Spike titers in cord blood were significantly more likely to have a longer infection-free interval than infants with lower titers (Figure 1B, *p* = 0.027). Even when evaluating the total infant cohort, including those who did and who did not develop COVID-19, higher anti-Spike titers in cord blood suggested longer interval to SARS-CoV-2 infection in infants, although not unexpectedly, this protection was lost over time (Figure A2). Notably, all infants with detectable cord blood titers remained infection-free until 3.5 months of age.

**Figure 2:**
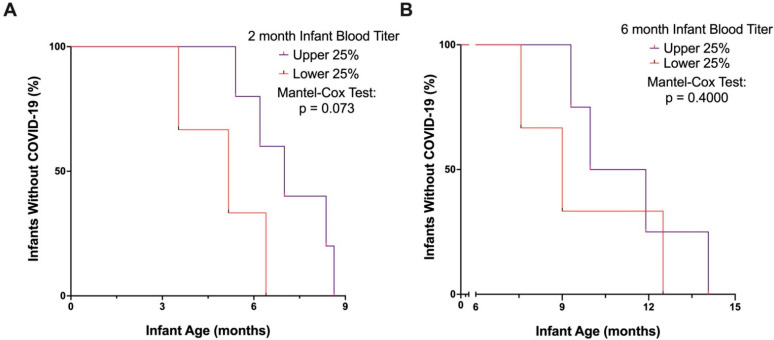
Relationship between infant 2-month and 6-month antibody titers and SARS-CoV-2 infection. Anti-Spike titers were measured in two- and six-month-old infants who were born to COVID-19 vaccinated individuals. (**A**) Kaplan–Meier curve displays time to SARS-CoV-2 infection for infants who developed COVID-19 through 9 months of age, grouped by those with high (upper 25%) and low (lower 25%) anti-Spike IgG titers in blood at two months of age (**B**) Kaplan–Meier curve displays time to SARS-CoV-2 infection for infants who developed COVID-19 after six months of age, grouped by those with high (upper 25%) and low (lower 25%) anti-Spike IgG titers in blood at six months of age. Analyses performed by a Mantel-Cox test.

We then sought to determine the durability of protection by passive transfer of maternal vaccine-generated antibodies against SARS-CoV-2 in infants, knowing that antibody levels decline considerably over time. First, we aimed to determine whether differences in antibody titers detected at two months of age were associated with interval to SARS-CoV-2 infection. Of those infants who tested positive for SARS-CoV-2 before 9 months of age, those with the highest levels of anti-Spike IgG titers at two months of age may be more likely to have a longer infection-free interval than infants with lower titers, although this finding did not achieve statistical significance (Figure 2A, *p* = 0.073). However, beyond 9 months of age, there was no significant relationship between 2-month antibody levels and infection-free interval, likely due to significant waning of antibody-mediated protection from maternally transferred antibodies by that age. Importantly, all infants with the highest quartile of titers at two months of age remained infection-free until five months of age.

**Figure A1:**
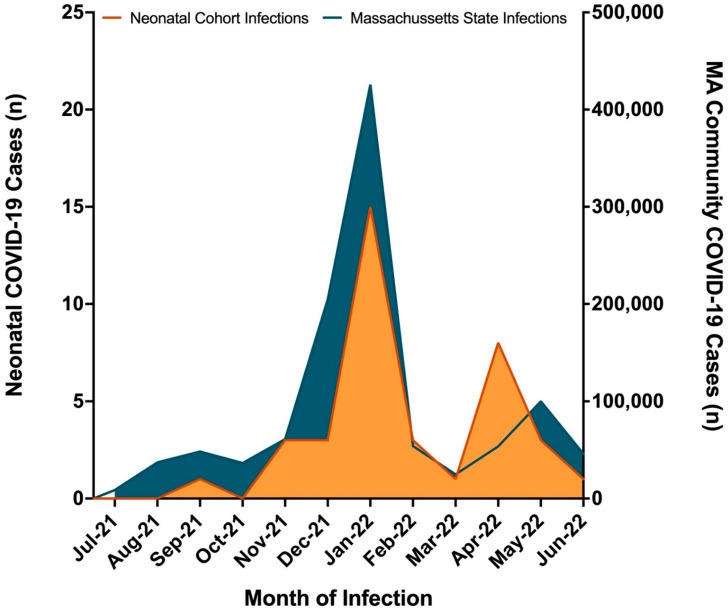
Cases of neonatal COVID-19 in the context of community case rates. Number of reported community SARS-CoV-2 infections in Massachusetts (MA) (right axis) and reports of infant infections in our infant cohort (left axis). Infant cases of COVID-19 in our study align with cases in MA community, suggesting community spread contributes to an infant’s susceptibility to SARS-CoV-2 infection. MA community COVID-19 case data extracted from MA Department of Public Health [27].

**Figure A2:**
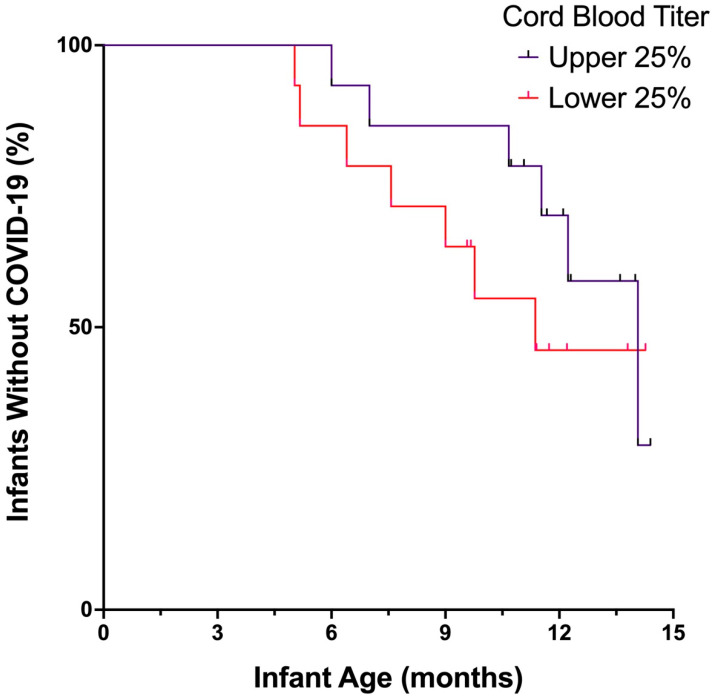
Relationship between cord blood antibody titers and SARS-CoV-2 infection in whole infant cohort. Kaplan–Meier curve displays time to SARS-CoV-2 infection of all infants in the cohort with cord antibody titers in the upper and lower quartiles, including those who developed COVID-19 and those who remained infection-free. Cord blood anti-Spike IgG titers in the top quartile suggested longer COVID-19 disease-free interval, although this finding did not achieve statistical significance over the 15-month follow up period (Mantel-Cox *p* = 0.333).

Given the degree to which infants’ anti-Spike titers wane by six months of age, we did not expect passive immunity to provide substantial protection against SARS-CoV-2 infection after six months. Consistent with this expectation, the vast majority of SARS-CoV-2 positive cases were reported in infants after six months of age, although timing of infection in the infant cohort was also influenced by the emergence of the highly transmissible Omicron variant of concern (VOC). As expected, SARS-CoV-2 case frequency in our infant cohort reflected community test-positive rates, which rose substantially with the emergence of Omicron (Figure A1). We sought to analyze duration of time (in months) until infant SARS-CoV-2 infection after six months of age, comparing infants who had the highest quartile of anti-Spike IgG at six months with those who had antibody levels in the lowest quartile. Infants with a SARS-CoV-2 infection prior to six months of age were excluded from this analysis. As expected, infant six-month antibody levels did not have a significant association with time to SARS-CoV-2 infection from six to fifteen months of age (Figure 2b). This finding suggests that anti-Spike antibody levels passively transferred to the infant during pregnancy offer limited protection after six months of age.

## 4. Discussion

While a majority of infants born to individuals vaccinated against COVID-19 have detectable anti-SARS-CoV-2-Spike antibodies for up to six months of age [8], whether antibody levels correlate with protection in infants remains to be defined. Here, we show that transplacental transfer of vaccine-induced anti-Spike IgG from mother to fetus provides robust protection against SARS-CoV-2 infection for the first few months of infancy, and that cord blood antibody levels at birth are significantly associated with longer disease-free interval in the infant. Higher antibody levels at two months also favored a longer disease-free interval through nine months of age.

Achieving high levels of anti-Spike IgG in the neonate (through transplacental transfer during pregnancy) appears to be key in minimizing susceptibility to SARS-CoV-2 infection in infants. In our cohort, no infants with detectable anti-Spike IgG at birth developed SARS-CoV-2 infection before 3.5 months of age. It has been shown that maternal vaccination achieves antibody titers in cord blood that far exceed titers gained from natural infection [18], and timing of vaccination in pregnancy and type of COVID-19 vaccine impact transferred antibody levels [5]. Maternal boosting in the third trimester has also been shown to produce substantial transplacental transfer of maternal IgG, with highest cord titers achieved when boosting occurred at least 60 days prior to delivery [21]. Thus, there exist strong rationale for vaccination in pregnancy beyond the primary focus of protecting pregnant individuals from severe COVID-19 [18,22,23]. This underscores the importance of optimizing SARS-CoV-2 vaccination and boosting strategies in pregnancy to allow for the highest transfer of anti-SARS-CoV-2 antibodies to infants. Given the small number of cases of vaccinated and infected individuals (n = 6) in our cohort, we did not explore how both maternal SARS-CoV-2 infection during pregnancy as well as maternal complete (two dose) COVID-19 vaccination in pregnancy impacted transplacental transfer of anti-Spike antibodies to infants and consequently protection from infant SARS-CoV-2 infection. Larger studies aimed at testing strategies to boost maternal transfer of anti-SARS-CoV-2 antibodies and improve effectiveness of maternally transferred antibodies are needed. 

All infants, regardless of antibody level at birth, displayed a steady decrease in anti-Spike IgG levels over the first six months of life, and protection against SARS-CoV-2 infection was also noted to wane significantly. This highlights the need for vaccinating infants by six months of age to boost protection against infection and severe disease. Fortunately, both the Pfizer (*BNT162b2*) and the Moderna (*mRNA-1273*) COVID-19 vaccines have recently been approved for use by the US FDA in children six months of age and older. The data presented here suggest that delaying vaccination in infants beyond 6 months of age will leave infants vulnerable to infection even if born to vaccinated mothers. These findings provide additional rationale for pediatricians and other healthcare providers to urge families to vaccinate infants as soon as they are eligible.

Waning immunity in the setting of novel variants compounds vulnerability to COVID-19. In our study, there was a sharp increase in the number of cases of infant SARS-CoV-2 during the Omicron epoch, mirroring the relatively high burden of SARS-CoV-2 positivity in the Massachusetts community due to the highly transmissible Omicron variant. Thus, waning immunity from passively transferred maternal antibody is not the only factor to consider when evaluating infant risk for COVID-19; community test-positive rates, and transmissibility and severity of VOCs are other key factors to consider when evaluating infant risk. While optimizing neonatal and infant antibody levels may provide protection, additional strategies including indoor masking, avoiding crowds, and “cocooning” (i.e., ensuring all close contacts of the infant are vaccinated against COVID-19) also remain key public health strategies to protect infants against disease.

While these data reflect a limited cohort study of serologic responses and parent-reported SARS-CoV-2 infection outcomes, they are the first data linking infant antibody levels with protection against SARS-CoV-2 infection in the first few months of life. Not all infant-mother dyads or triads were able to provide samples at each time point due to clinical restrictions at time of collection or parental choice. Despite research demonstrating the efficacy of COVID-19 vaccines in pregnancy [24], vaccine hesitancy is a multifactorial problem that persists amongst pregnant people and represents a significant health concern for this population [25,26], thus there may be some bias in individuals who participated in our study. It is possible that mothers who received COVID-19 vaccination in pregnancy might be more likely to implement risk-mitigation strategies such as masking and social distancing that could also reduce infant risk of contracting SARS-CoV-2, but these factors were not assessed in this study.

Eighty-five percent of our maternal cohort identified as “White”, a majority of whom were non-Hispanic. The enrichment of our cohort for White, non-Hispanic patients may limit the generalizability of our results to more diverse cohorts with different risk factors for COVID-19 exposure and/or vaccination status. In addition, our study did not evaluate COVID-19 outcomes in infants born to unvaccinated mothers. While protective benefits of maternal COVID-19 vaccination in pregnancy against infant hospitalization prior to 6 months of age have previously been shown in a large cohort [6,7], antibody titers of those infants were unknown. Of note, nearly 50% of infant COVID-19 cases were not obtained by laboratory analyzed PCR testing and therefore not tracked by state level COVID-19 tracking, highlighting the fact that infant and pediatric cases are likely highly underreported by usual data-gathering measures. We did not include routine SARS-CoV-2 screening in our study; thus, it is possible cases of COVID-19 in the infants were missed.

## 5. Conclusions

In summary, maternally transferred anti-Spike IgG is protective against SARS-CoV-2 infection in infants, with the highest levels in cord blood at birth being significantly correlated with longer infection-free intervals in infants. By six months of age, antibody levels and antibody-mediated protection have waned, and infant vaccination is warranted. Given the evolving nature of SARS-CoV-2 and future VOCs that may emerge in the months to come, greater understanding of the neonatal immune profile after maternal vaccination in pregnancy is needed. Our study addresses a key knowledge gap between detectable anti-SARS-CoV-2 titers in infants and antibody-mediated protection against COVID-19.

## Figures and Tables

**Table 1:** Demographics of the vaccinated maternal cohort and their infants. To assess for differences between SARS-CoV-2 infected and uninfected neonatal cohorts, continuous outcomes were analyzed by Mann–Whitney U test and dichotomous outcomes were analyzed by Fisher’s Exact test. The maternal cohort was n = 105 and the neonatal cohort was n = 107 due to two sets of twins.

Maternal Cohort Characteristics	Vaccinated (n = 105)		
Maternal Age at Sample Collection, mean (SD), years	34.6 (3.3)		
Gestational Age at Vaccination, mean (SD), weeks	25.8 (7.4)		
Maternal Vaccine Platform, number (%)			
BNT162b2	62 (59.0)		
mRNA-1273	34 (32.4)		
Ad26.COV2.S	9 (8.6)		
SARS-CoV-2 Infection During Pregnancy, number (%)	6 (5.7)		
Gestational Age at SARS-CoV-2 Infection, mean (SD), weeks	17.5 (13.6)		
Hispanic, number (%)	6 (5.7)		
Race, number (%)			
Asian	7 (6.7)		
Black	3 (2.9)		
Other	3 (2.9)		
White	90 (85.7)		
Unknown	2 (1.9)		
**Neonatal Cohort Characteristics**	**SARS-CoV-2 Infected (n = 38)**	**Uninfected (n = 69)**	***p***
Male sex, number (%)	20 (52.6)	33 (47.8)	0.634
Gestational Age at Delivery, mean (SD), weeks	39.1 (1.0)	39.0 (1.6)	0.983
Birthweight, mean (SD), g	3422 (477)	3237 (550)	0.053
Breastfed ^#^, number (%)	24 (63.2)	42 (60.9)	0.839

^#^ Breastfeeding status was determined at time of SARS-CoV-2 infection for the infected infant cohort and at study completion for the uninfected infant cohort.

**Table 2:** Presentation and clinical outcomes of infants infected with SARS-CoV-2 who were born to individuals vaccinated in pregnancy.

Clinical Characteristics of Infants with COVID-19	Infants (n = 38)
Age at SARS-CoV-2 Infection, average (SD), months	8.5 (3.0)
COVID-19-Related Hospitalizations, n (%)	0
Symptoms Reported, n (%)	
Congestion/rhinorrhea	33 (86.8)
Cough	25 (65.8)
Fever	22 (57.9)
Increased fussiness	7 (18.4)
Parent perception of fatigue	6 (15.8)
Parent perception of reduced appetite	3 (7.9)
Parent perception of difficulty swallowing	2 (5.3)
Parent perception of labored breathing	2 (5.3)
Parent perception of difficulty sleeping	2 (5.3)
Vomiting	2 (5.3)
Diarrhea	2 (5.3)
Rash	2 (5.3)
At-Home Treatments Reported, n (%)	
Antipyretics	18 (47.4)
Oral Steroids for wheezing	2 (5.3)
None	18 (47.4)
Testing Modality, number (%)	
PCR	21 (55.3)
Rapid Antigen	15 (39.5)
Never tested, but exposed and symptomatic	2 (5.3)

## Data Availability

Not applicable.
